# Correction: Derivation and validation of an algorithm to predict transitions from community to residential long-term care among persons with dementia—A retrospective cohort study

**DOI:** 10.1371/journal.pdig.0000987

**Published:** 2025-08-13

**Authors:** Wenshan Li, Luke Turcotte, Amy T. Hsu, Robert Talarico, Danial Qureshi, Colleen Webber, Steven Hawken, Peter Tanuseputro, Douglas G. Manuel, Greg Huyer

There is an error in affiliation 2 for author Luke Turcotte. The correct affiliation 2 is: Brock University, Department of Health Sciences, St. Catherines, Ontario, Canada.
