# Supplementary material for: Derivation and validation of an algorithm to predict transitions from community to residential long-term care among persons with dementia—A retrospective cohort study
Source: PLOS Digit Health. 2024 Oct 18;3(10):e0000441. doi: 10.1371/journal.pdig.0000441 (PMC11488705; doi:10.1371/journal.pdig.0000441)
Supplement: S1 Table — (DOCX) [file pdig.0000441.s002.docx]

**S1. Table –** Comprehensive Characteristics of the total, derivation, and validation cohorts

|  |  | **n (%)** | | |
| --- | --- | --- | --- | --- |
| **Characteristics of Individuals with Dementia** | | **Total Cohort n=129,851** | **Derivation Cohort n=95,813** | **Validation Cohort n=34,038** |
| Sex | Female | 79,334 (61.1%) | 58,859 (61.4%) | 20,475 (60.2%) |
|  | Male | 50,517 (38.9%) | 36,954 (38.6%) | 13,563 (39.8%) |
| Age | Mean ± SD | 83.7 ± 7.08 | 83.6 ± 7.00 | 84.0 ± 7.31 |
|  | Median (IQR) | 84 (79-89) | 84 (79-89) | 85 (79-89) |
| Marital status | Married | 49,659 (38.2%) | 36,982 (38.6%) | 12,677 (37.2%) |
|  | Widowed | 64,546 (49.7%) | 47,727 (49.8%) | 16,819 (49.4%) |
|  | Separated or divorced | 8,745 (6.7%) | 6,142 (6.4%) | 2,603 (7.6%) |
|  | Never married | 5,452 (4.2%) | 3,930 (4.1%) | 1,522 (4.5%) |
|  | Other | 1,449 (1.1%) | 1,032 (1.1%) | 417 (1.2%) |
| Education | Grade 11 or lower | 33,491 (25.8%) | 25,283 (26.4%) | 8,208 (24.1%) |
|  | High school | 15,822 (12.2%) | 11,765 (12.3%) | 4,057 (11.9%) |
|  | Technical or trade school | 6,080 (4.7%) | 4,489 (4.7%) | 1,591 (4.7%) |
|  | Some college, university, diploma/bachelor's degree, or graduate degree | 15,601 (12.0%) | 11,378 (11.9%) | 4,223 (12.4%) |
|  | Unknown | 58,857 (45.3%) | 42,898 (44.8%) | 15,959 (46.9%) |
| Time since last hospital stay | No hospitalization in 180 days | 40,759 (31.4%) | 30,705 (32.0%) | 10,054 (29.5%) |
|  | Presently in hospital | 21,207 (16.3%) | 16,803 (17.5%) | 4,404 (12.9%) |
|  | Within the last 30 days | 21,458 (16.5%) | 14,878 (15.5%) | 6,580 (19.3%) |
|  | More than 30 days ago | 10,124 (7.8%) | 7,267 (7.6%) | 2,857 (8.4%) |
|  | Missing | 36,303 (28.0%) | 26,160 (27.3%) | 10,143 (29.8%) |
| Person lives with others | | 13,758 (10.6%) | 9,967 (10.4%) | 3,791 (11.1%) |
| Had access to home | | 13,511 (10.4%) | 8,888 (9.3%) | 4,623 (13.6%) |
| Had access to rooms in house | | 10,264 (7.9%) | 6,769 (7.1%) | 3,495 (10.3%) |
| LHIN | Missing | 47 (0.0%) | 38 (0.0%) | 8 (0.0%) |
|  | Erie St. Clair | 6,646 (5.1%) | 4,970 (5.2%) | 1,677 (4.9%) |
|  | South West | 10,777 (8.3%) | 7,951 (8.3%) | 2,828 (8.3%) |
|  | Waterloo Wellington | 6,991 (5.4%) | 5,128 (5.4%) | 1,860 (5.5%) |
|  | Hamilton Niagara Haldimand Brant | 16,327 (12.6%) | 12,066 (12.6%) | 4,280 (12.6%) |
|  | Central West | 4,672 (3.6%) | 3,448 (3.6%) | 1,221 (3.6%) |
|  | Mississauga Halton | 8,249 (6.4%) | 6,155 (6.4%) | 2,075 (6.1%) |
|  | Toronto Central | 10,844 (8.4%) | 8,089 (8.4%) | 2,796 (8.2%) |
|  | Central | 14,681 (11.3%) | 10,724 (11.2%) | 3,955 (11.6%) |
|  | Central East | 16,206 (12.5%) | 11,780 (12.3%) | 4,386 (12.9%) |
|  | South East | 6,597 (5.1%) | 4,832 (5.0%) | 1,756 (5.2%) |
|  | Champlain | 12,416 (9.6%) | 9,233 (9.6%) | 3,189 (9.4%) |
|  | North Simcoe Muskoka | 5,251 (4.0%) | 3,937 (4.1%) | 1,315 (3.9%) |
|  | North East | 7,321 (5.6%) | 5,387 (5.6%) | 1,942 (5.7%) |
|  | North West | 2,826 (2.2%) | 2,075 (2.2%) | 750 (2.2%) |
| **Functional and Health Status** | |  |  |  |
| ADL Self-Performance Hierarchy | 0: Independent | 48,019 (37.0%) | 36,326 (37.9%) | 11,693 (34.4%) |
|  | 1: At least supervision in 1 ADL (less than limited in all 4) | 21,321 (16.4%) | 15,654 (16.3%) | 5,667 (16.6%) |
|  | 2: Limited assistance in 1+ ADLs (less than extensive in all 4) | 25,734 (19.8%) | 18,594 (19.4%) | 7,140 (21.0%) |
|  | 3: At least extensive assistance in personal hygiene or toilet use (less than extensive in eating and locomotion) | 14,008 (10.8%) | 9,851 (10.3%) | 4,157 (12.2%) |
|  | 4: Extensive assistance in eating or locomotion (total dependence in neither) | 11,077 (8.5%) | 8,269 (8.6%) | 2,808 (8.2%) |
|  | 5: Total dependence in eating and/or locomotion | 7,817 (6.0%) | 5,702 (6.0%) | 2,115 (6.2%) |
|  | 6: Total dependence in all 4 ADLs | 1,875 (1.4%) | 1,417 (1.5%) | 458 (1.3%) |
| IADL Performance Scale | 0: Independent | 1,577 (1.2%) | 1,219 (1.3%) | 358 (1.1%) |
|  | 1: Setup help only | 2,391 (1.8%) | 1,846 (1.9%) | 545 (1.6%) |
|  | 2: Supervision | 7,431 (5.7%) | 5,740 (6.0%) | 1,691 (5.0%) |
|  | 3: Limited assistance | 3,784 (2.9%) | 2,932 (3.1%) | 852 (2.5%) |
|  | 4: Extensive assistance | 15,482 (11.9%) | 11,486 (12.0%) | 3,996 (11.7%) |
|  | 5: Maximal assistance | 54,037 (41.6%) | 39,255 (41.0%) | 14,782 (43.4%) |
|  | 6: Total dependence | 45,149 (34.8%) | 33,335 (34.8%) | 11,814 (34.7%) |
| Incontinence worsened |  | 33,680 (25.9%) | 24,325 (25.4%) | 9,355 (27.5%) |
| CHESS Score | 0: No health instability | 18,159 (14.0%) | 14,441 (15.1%) | 3,718 (10.9%) |
|  | 1: Minimal health instability | 32,017 (24.7%) | 24,440 (25.5%) | 7,577 (22.3%) |
|  | 2: Low health instability | 43,672 (33.6%) | 32,321 (33.7%) | 11,351 (33.3%) |
|  | 3: Moderate health instability | 24,268 (18.7%) | 16,864 (17.6%) | 7,404 (21.8%) |
|  | 4: High health instability | 11,273 (8.7%) | 7,448 (7.8%) | 3,825 (11.2%) |
|  | 5: Very high health instability | 462 (0.4%) | 299 (0.3%) | 163 (0.5%) |
| Cognitive Performance Scale | 0: Intact | 4,010 (3.1%) | 3,133 (3.3%) | 1. .6%) |
|  | 1: Borderline intact | 7,244 (5.6%) | 5,425 (5.7%) | 1,819 (5.3%) |
|  | 2: Mild impairment | 77,413 (59.6%) | 56,169 (58.6%) | 21,244 (62.4%) |
|  | 3: Moderate impairment | 27,278 (21.0%) | 20,349 (21.2%) | 6,929 (20.4%) |
|  | 4: Moderately severe impairment | 3,882 (3.0%) | 3,050 (3.2%) | 832 (2.4%) |
|  | 5: Severe impairment | 8,552 (6.6%) | 6,560 (6.8%) | 1,992 (5.9%) |
|  | 6: Very severe impairment | 1,472 (1.1%) | 1,127 (1.2%) | 345 (1.0%) |
| Depression Rating Scale | 0: No symptoms | 61,913 (47.7%) | 46,762 (48.8%) | 15,151 (44.5%) |
|  | 1 | 18,531 (14.3%) | 13,745 (14.3%) | 4,786 (14.1%) |
|  | 2 | 18,002 (13.9%) | 13,062 (13.6%) | 4,940 (14.5%) |
|  | 3+: Possible mood disturbance | 31,405 (24.2%) | 22,244 (23.2%) | 9,161 (26.9%) |
| Had delusions |  | 6,402 (4.9%) | 4,612 (4.8%) | 1,790 (5.3%) |
| Had hallucinations |  | 8,636 (6.7%) | 6,250 (6.5%) | 2,386 (7.0%) |
| Falls | 0 | 63,686 (49.0%) | 48,117 (50.2%) | 15,569 (45.7%) |
|  | 1 | 30,701 (23.6%) | 22,419 (23.4%) | 8,282 (24.3%) |
|  | 2 | 15,526 (12.0%) | 11,079 (11.6%) | 4,447 (13.1%) |
|  | 3+ | 19,938 (15.4%) | 14,198 (14.8%) | 5,740 (16.9%) |
| **Disease Diagnoses** | |  |  |  |
| Renal failure |  | 9,436 (7.3%) | 6,634 (6.9%) | 2,802 (8.2%) |
| Stroke |  | 24,695 (19.0%) | 18,099 (18.9%) | 6,596 (19.4%) |
| Congestive heart failure |  | 15,046 (11.6%) | 10,852 (11.3%) | 4,194 (12.3%) |
| Coronary heart disease |  | 31,140 (24.0%) | 22,798 (23.8%) | 8,342 (24.5%) |
| Peripheral vascular disease | | 7,223 (5.6%) | 5,136 (5.4%) | 2,087 (6.1%) |
| Hemiplegia/hemiparesis |  | 2,100 (1.6%) | 1,424 (1.5%) | 676 (2.0%) |
| Multiple sclerosis |  | 364 (0.3%) | 212 (0.2%) | 152 (0.4%) |
| Parkinsonism |  | 7,275 (5.6%) | 5,314 (5.5%) | 1,961 (5.8%) |
| Hip fracture |  | 6,687 (5.1%) | 4,878 (5.1%) | 1,809 (5.3%) |
| Other fractures |  | 10,238 (7.9%) | 7,411 (7.7%) | 2,827 (8.3%) |
| Any psychiatric diagnosis | | 22,454 (17.3%) | 16,074 (16.8%) | 6,380 (18.7%) |
| Cancer, not including skin cancer | | 12,158 (9.4%) | 8,745 (9.1%) | 3,413 (10.0%) |
| Emphysema/COPD/Asthma | | 18,510 (14.3%) | 13,230 (13.8%) | 5,280 (15.5%) |
| **Behavioural Symptoms (in last 3 days)** | |  |  |  |
| Wandered |  | 9,126 (7.0%) | 7,045 (7.4%) | 2,081 (6.1%) |
| Was verbally abusive |  | 10,285 (7.9%) | 7,718 (8.1%) | 2,567 (7.5%) |
| Was physically abusive |  | 2,828 (2.2%) | 2,191 (2.3%) | 637 (1.9%) |
| Was socially inappropriate or disruptive | | 5,601 (4.3%) | 4,285 (4.5%) | 1,316 (3.9%) |
| Resisted care |  | 17,844 (13.7%) | 13,311 (13.9%) | 4,533 (13.3%) |
| Behavioural symptoms changed | | 21,548 (16.6%) | 15,837 (16.5%) | 5,711 (16.8%) |
| **Health Services Utilization** | |  |  |  |
| Had home health aides, homemaking or meals services in last 7 days | No services | 65,688 (50.6%) | 49,322 (51.5%) | 16,366 (48.1%) |
|  | Services received | 35,279 (27.2%) | 25,909 (27.0%) | 9,370 (27.5%) |
|  | Missing | 28,884 (22.2%) | 20,582 (21.5%) | 8,302 (24.4%) |
| Had visiting nurses in last 7 days | No services | 106,978 (82.4%) | 79,435 (82.9%) | 27,543 (80.9%) |
|  | Services received | 13,582 (10.5%) | 9,309 (9.7%) | 4,273 (12.6%) |
|  | Missing | 9,291 (7.2%) | 7,069 (7.4%) | 2,222 (6.5%) |
| Had physical, occupational, or speech therapy in last 7 days | No services | 99,430 (76.6%) | 75,249 (78.5%) | 24,181 (71.0%) |
|  | Services received | 30,421 (23.4%) | 20,564 (21.5%) | 9,857 (29.0%) |
| Had day care/hospital, or social worker in home in last 7 days | No services | 125,878 (96.9%) | 92,938 (97.0%) | 32,940 (96.8%) |
|  | Services received | 3,973 (3.1%) | 2,875 (3.0%) | 1,098 (3.2%) |
| # of hospital admissions in last 90 days | No visits | 73,050 (56.3%) | 54,599 (57.0%) | 18,451 (54.2%) |
|  | 1 visit | 48,012 (37.0%) | 35,048 (36.6%) | 12,964 (38.1%) |
|  | 2 visits | 7,120 (5.5%) | 5,022 (5.2%) | 2,098 (6.2%) |
|  | 3+ visits | 1,669 (1.3%) | 1,144 (1.2%) | 525 (1.5%) |
| # of ER visits in last 90 days | No visits | 99,436 (76.6%) | 74,211 (77.5%) | 25,225 (74.1%) |
|  | 1 visit | 22,083 (17.0%) | 15,883 (16.6%) | 6,200 (18.2%) |
|  | 2 visits | 5,408 (4.2%) | 3,747 (3.9%) | 1,661 (4.9%) |
|  | 3+ visits | 2,924 (2.3%) | 1,972 (2.1%) | 952 (2.8%) |
| Number of medications | 0 to 8 | 65,424 (50.4%) | 47,724 (49.8%) | 17,700 (52.0%) |
|  | 9 or more | 64,427 (49.6%) | 48,089 (50.2%) | 16,338 (48.0%) |
| Received antipsychotic/neuroleptic medication | | 20,657 (15.9%) | 16,030 (16.7%) | 4,627 (13.6%) |
| Received anxiolytic medication | | 18,485 (14.2%) | 14,219 (14.8%) | 4,266 (12.5%) |
| Received antidepressant medication | | 36,698 (28.3%) | 27,099 (28.3%) | 9,599 (28.2%) |
| Received hypnotic medication | | 40,175 (30.9%) | 28,026 (29.3%) | 12,149 (35.7%) |
| **Caregiving/Caregiver Characteristics** | |  |  |  |
| Primary caregiver lives with person | | 66,098 (50.9%) | 48,529 (50.6%) | 17,569 (51.6%) |
| Primary caregiver's relationship with person | Child or child-in-law | 73,066 (56.3%) | 53,746 (56.1%) | 19,320 (56.8%) |
|  | Spouse | 38,348 (29.5%) | 28,781 (30.0%) | 9,567 (28.1%) |
|  | Other relative | 10,334 (8.0%) | 7,570 (7.9%) | 2,764 (8.1%) |
|  | Friend or neighbor | 5,795 (4.5%) | 4,342 (4.5%) | 1,453 (4.3%) |
|  | Missing | 2,308 (1.8%) | 1,374 (1.4%) | 934 (2.7%) |
| Primary caregiver unable to continue | | 26,765 (20.6%) | 18,299 (19.1%) | 8,466 (24.9%) |
| Primary caregiver not satisfied with support from family | | 9,930 (7.6%) | 6,029 (6.3%) | 3,901 (11.5%) |
| Primary caregiver felt distress, anger, or depression | | 46,011 (35.4%) | 31,804 (33.2%) | 14,207 (41.7%) |
| Hours of informal care | 0 hours | 26,743 (20.6%) | 20,621 (21.5%) | 6,122 (18.0%) |
|  | 1 to 24 hours | 67,676 (52.1%) | 49,324 (51.5%) | 18,352 (53.9%) |
|  | 25 to 48 hours | 24,698 (19.0%) | 18,053 (18.8%) | 6,645 (19.5%) |
|  | Greater than 48 hours | 10,734 (8.3%) | 7,815 (8.2%) | 2,919 (8.6%) |
