# Supplementary material for: Derivation and validation of an algorithm to predict transitions from community to residential long-term care among persons with dementia—A retrospective cohort study
Source: PLOS Digit Health. 2024 Oct 18;3(10):e0000441. doi: 10.1371/journal.pdig.0000441 (PMC11488705; doi:10.1371/journal.pdig.0000441)
Supplement: S2 Table — (DOCX) [file pdig.0000441.s003.docx]

**S2.Table** – Full regression estimates for the total cohort

|  |  | **Hazard Ratio** | **95% Confidence Interval** | |
| --- | --- | --- | --- | --- |
| Sex (reference: female) | Male | 0.884 | 0.861 | 0.907 |
| Age | 1st segment of the  restricted cubic spline (RCS) | 1.014 | 1.007 | 1.021 |
|  | 2nd segment of RCS | 0.964 | 0.940 | 0.990 |
|  | 3rd segment of RCS | 1.362 | 1.113 | 1.667 |
|  | 4th segment of RCS | 0.558 | 0.384 | 0.811 |
| Marital status  (Reference: married) | Widowed | 1.144 | 1.100 | 1.190 |
|  | Separated or divorced | 1.232 | 1.166 | 1.301 |
|  | Never married | 1.188 | 1.111 | 1.271 |
|  | Other | 1.127 | 1.014 | 1.253 |
| Education  (reference: Grade 11 or lower) | High school | 0.985 | 0.947 | 1.024 |
|  | Technical or trade school | 1.030 | 0.974 | 1.090 |
|  | Some college, university, or graduate degree | 0.877 | 0.841 | 0.914 |
|  | Unknown | 0.910 | 0.882 | 0.939 |
| Time since last hospital stay  (reference: no hospitalization in last 180 days) | Presently in hospital | 1.125 | 1.077 | 1.176 |
|  | Within the last 30 days | 0.867 | 0.828 | 0.908 |
|  | >30 days ago | 0.743 | 0.705 | 0.783 |
|  | Missing | 1.142 | 1.043 | 1.249 |
| Place of residence at time of referral (reference: private home) | Board care, assisted living, group home, or residential care facility | 1.218 | 1.171 | 1.268 |
|  | Other | 1.114 | 1.023 | 1.212 |
| ADL Self-Performance Hierarchy score (reference: score 0) | 1 | 1.141 | 1.102 | 1.183 |
|  | 2 | 1.252 | 1.209 | 1.297 |
|  | 3 | 1.373 | 1.316 | 1.433 |
|  | 4 | 1.390 | 1.324 | 1.459 |
|  | 5 | 1.332 | 1.260 | 1.408 |
|  | 6 | 0.952 | 0.834 | 1.086 |
| IADL Score  (reference: score 0) | 1 | 1.209 | 1.008 | 1.449 |
|  | 2 | 1.348 | 1.152 | 1.577 |
|  | 3 | 1.884 | 1.603 | 2.215 |
|  | 4 | 1.735 | 1.492 | 2.017 |
|  | 5 | 2.161 | 1.862 | 2.508 |
|  | 6 | 2.293 | 1.973 | 2.666 |
| CHESS Score  (reference: score 0) | 1 | 1.220 | 1.173 | 1.270 |
|  | 2 | 1.327 | 1.276 | 1.380 |
|  | 3 | 1.239 | 1.185 | 1.295 |
|  | 4 | 1.067 | 1.009 | 1.129 |
|  | 5 | 0.557 | 0.431 | 0.719 |
| Cognitive Performance Scale  (Reference: score 0) | 1 | 1.063 | 0.973 | 1.161 |
|  | 2 | 1.118 | 1.038 | 1.205 |
|  | 3 | 1.352 | 1.250 | 1.462 |
|  | 4 | 1.222 | 1.111 | 1.344 |
|  | 5 | 1.318 | 1.209 | 1.437 |
|  | 6 | 1.140 | 0.971 | 1.339 |
| Depression Rating Scale  (reference: score 0) | 1 | 1.036 | 1.002 | 1.071 |
|  | 2 | 1.033 | 0.999 | 1.069 |
|  | 3+ | 0.994 | 0.964 | 1.025 |
| Wandered (reference: no) | Yes | 1.333 | 1.280 | 1.389 |
| Was verbally abusive (reference: no) | Yes | 1.048 | 1.002 | 1.096 |
| Was physically abusive (reference: no) | Yes | 0.891 | 0.829 | 0.958 |
| Was socially inappropriate/disruptive (reference: no) | Yes | 1.028 | 0.977 | 1.081 |
| Resisted care (reference: no) | Yes | 1.011 | 0.977 | 1.046 |
| Behavioural symptoms changed (reference: no) | Yes | 1.124 | 1.087 | 1.163 |
| Primary caregiver lived with person (reference: no) | Yes | 0.775 | 0.752 | 0.799 |
| Primary caregiver's relationship to person  (reference: child or child-in-law) | Spouse | 0.909 | 0.869 | 0.950 |
|  | Other relative | 1.111 | 1.065 | 1.160 |
|  | Friend or neighbour | 1.030 | 0.975 | 1.087 |
|  | Missing | 0.725 | 0.663 | 0.794 |
| Primary caregiver unable to continue (reference: no) | Yes | 1.306 | 1.270 | 1.344 |
| Caregiver is not satisfied with support from family (reference: no) | Yes | 0.875 | 0.835 | 0.917 |
| Primary caregiver felt distress, anger, or depression (reference: no) | Yes | 1.167 | 1.138 | 1.197 |
| Informal hours of care per week (reference: 0 hour) | 1 to 24 hours | 0.474 | 0.452 | 0.497 |
|  | 25 to 48 hours | 0.469 | 0.445 | 0.494 |
|  | Greater than 48 hours | 0.483 | 0.455 | 0.512 |
| Incontinence worsened (reference: no) | Yes | 1.059 | 1.031 | 1.087 |
| Renal failure (reference: no) | Yes | 0.907 | 0.867 | 0.949 |
| Stroke (reference: no) | Yes | 1.026 | 0.997 | 1.056 |
| Congestive heart failure (reference: no) | Yes | 0.903 | 0.870 | 0.938 |
| Coronary heart disease (reference: no) | Yes | 0.997 | 0.970 | 1.024 |
| Peripheral vascular disease (reference: no) | Yes | 0.996 | 0.949 | 1.046 |
| Hemiplegia/hemiparesis (reference: no) | Yes | 0.918 | 0.838 | 1.005 |
| Parkinsonism (reference: no) | Yes | 1.001 | 0.953 | 1.052 |
| Hip fracture (reference: no) | Yes | 1.013 | 0.965 | 1.063 |
| Other fractures (reference: no) | Yes | 0.980 | 0.940 | 1.020 |
| Cancer (reference: no) | Yes | 0.914 | 0.878 | 0.952 |
| Emphysema/COPD/Asthma  (reference: no) | Yes | 0.945 | 0.914 | 0.978 |
| Delusions (reference: no) | Yes | 1.044 | 0.994 | 1.096 |
| Hallucinations (reference: no) | Yes | 1.089 | 1.042 | 1.138 |
| Falls Frequency (reference: no falls) | 1 | 1.061 | 1.031 | 1.091 |
|  | 2 | 1.180 | 1.138 | 1.223 |
|  | 3+ | 1.240 | 1.199 | 1.282 |
| Had access to home (reference: no) | Yes | 0.910 | 0.874 | 0.948 |
| Had access to rooms in house  (reference: no) | Yes | 0.898 | 0.853 | 0.944 |
| Person lives with others (reference: no) | Yes | 1.077 | 1.040 | 1.115 |
| Had home health aides, homemaking or meals services in last 7 days  (reference: did not receive service) | Services received | 0.806 | 0.780 | 0.833 |
|  | Missing | 0.898 | 0.866 | 0.931 |
| Had visiting nurses in last 7 days (reference: did not receive service) | Services received | 0.936 | 0.896 | 0.978 |
|  | Missing | 1.075 | 1.029 | 1.124 |
| Had physical, occupational, or speech therapy in last 7 days  (reference: did not receive service) | Services received | 0.891 | 0.863 | 0.919 |
| Number of hospital admissions in last 90 days (reference: 0) | 1 | 0.999 | 0.969 | 1.030 |
|  | 2 | 0.981 | 0.930 | 1.035 |
|  | 3+ | 0.942 | 0.848 | 1.046 |
| Number of emergency room visits in last 90 days  (reference: 0) | 1 | 1.012 | 0.982 | 1.043 |
|  | 2 | 1.083 | 1.023 | 1.145 |
|  | 3+ | 1.064 | 0.986 | 1.149 |
| Number of medications (reference: 0-8) | 9 or more | 0.964 | 0.940 | 0.988 |
| Received antipsychotic/neuroleptic medication (reference: no) | Yes | 1.019 | 0.989 | 1.050 |
| Received anxiolytic medication (reference: no) | Yes | 0.980 | 0.950 | 1.011 |
| Received antidepressant medication (reference: no) | Yes | 1.031 | 1.005 | 1.058 |
| Received hypnotic medication  (reference: no) | Yes | 1.015 | 0.990 | 1.041 |
