# Supplementary figures and images for: Derivation and validation of an algorithm to predict transitions from community to residential long-term care among persons with dementia—A retrospective cohort study
Source: PLOS Digit Health. 2024 Oct 18;3(10):e0000441. doi: 10.1371/journal.pdig.0000441 (PMC11488705; doi:10.1371/journal.pdig.0000441)

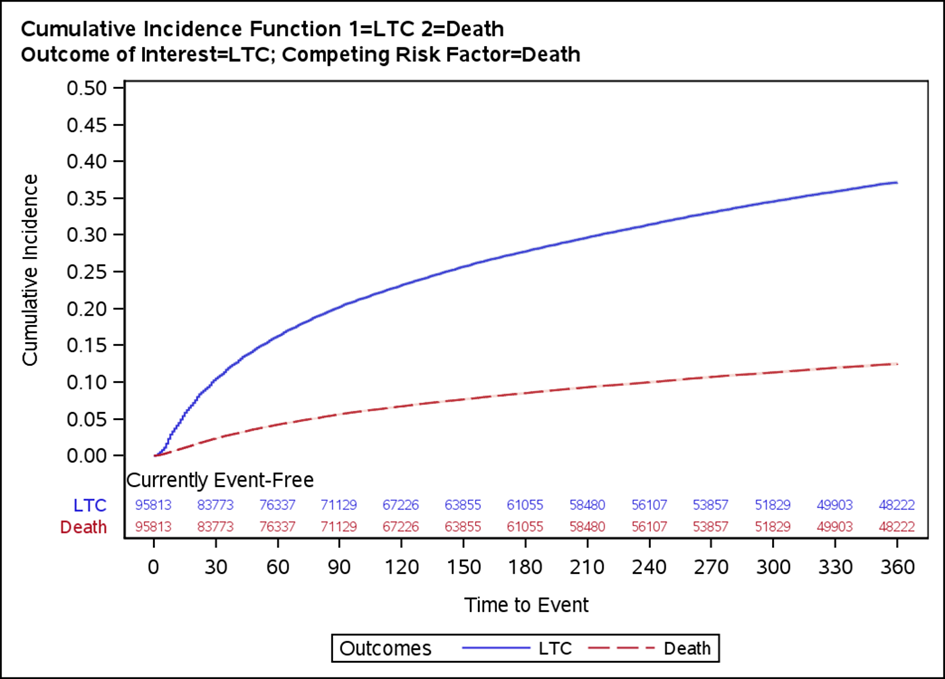

Supplement: S1 Fig — (TIF) [file pdig.0000441.s004.tif]

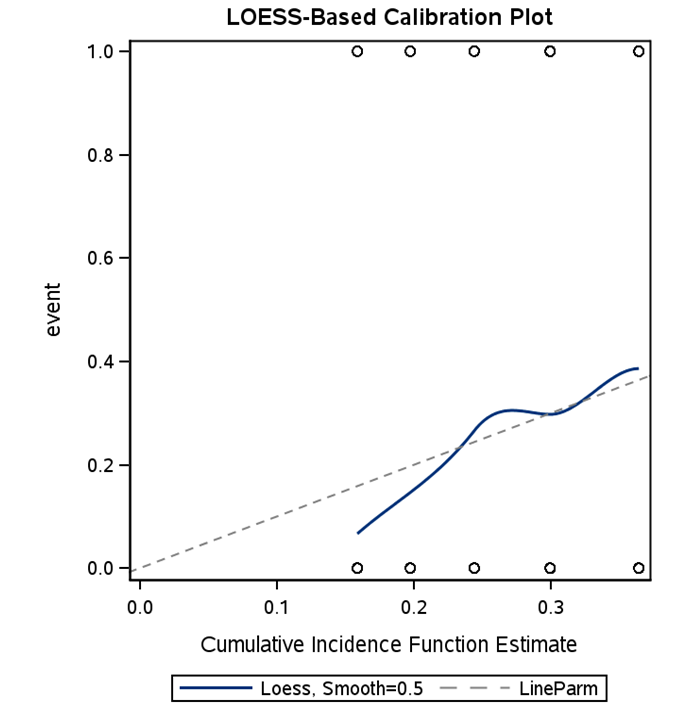

Supplement: S2 Fig — (TIF) [file pdig.0000441.s005.tif]

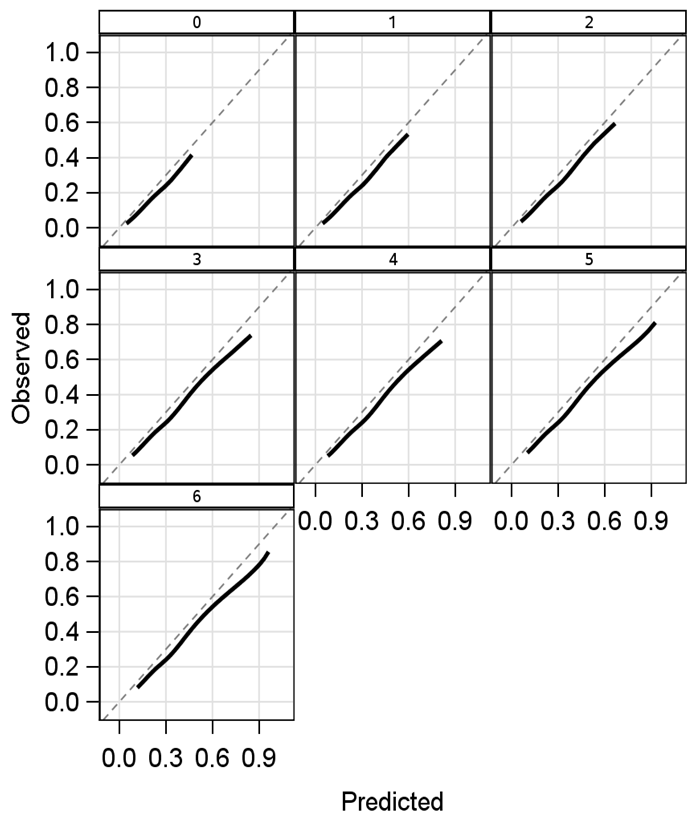

Supplement: S3 Fig — (TIF) [file pdig.0000441.s006.tif]

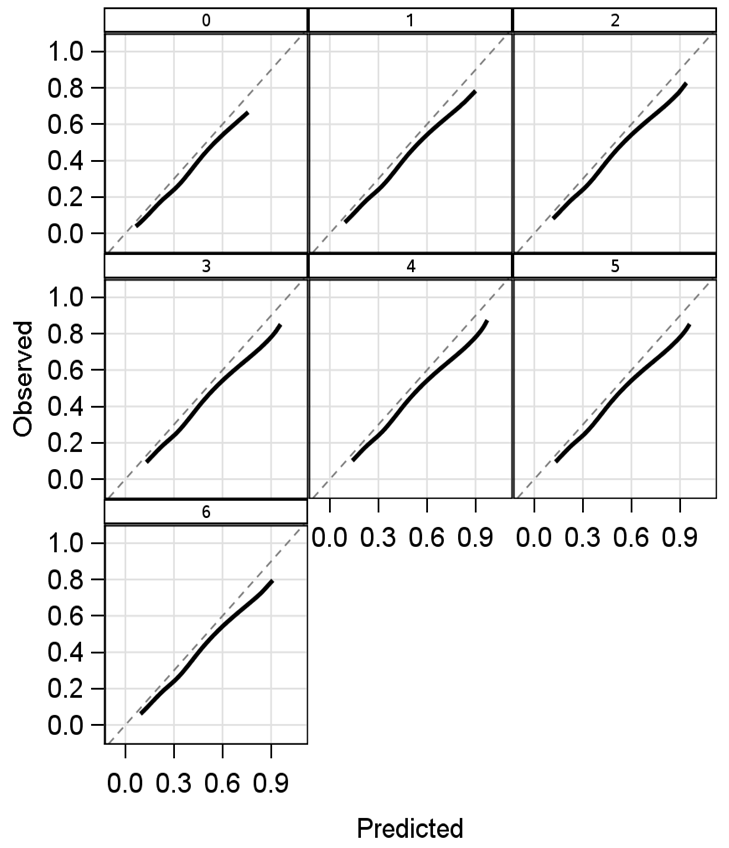

Supplement: S4 Fig — (TIF) [file pdig.0000441.s007.tif]

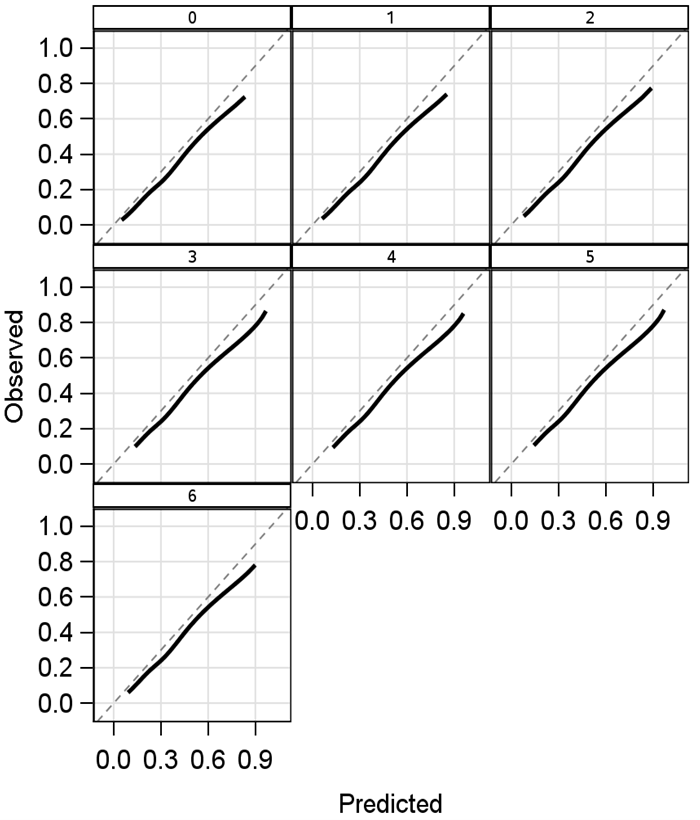

Supplement: S5 Fig — (TIF) [file pdig.0000441.s008.tif]

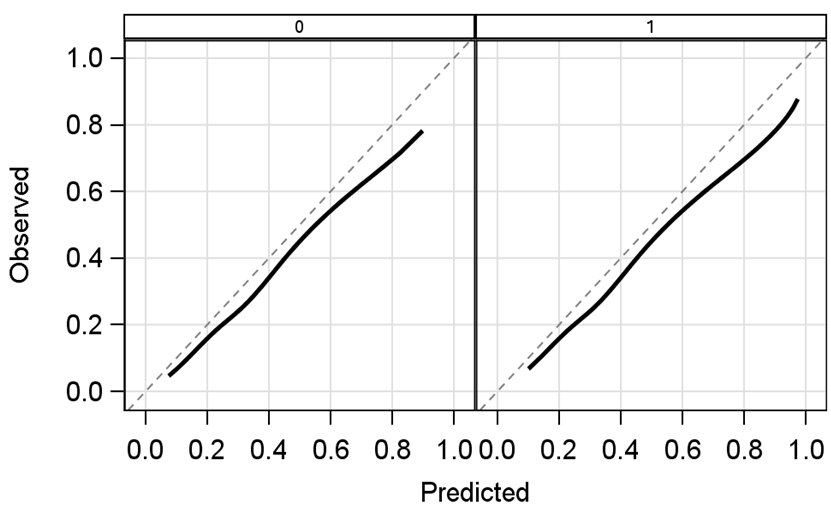

Supplement: S6 Fig — (TIF) [file pdig.0000441.s009.tif]
