# Supplementary material for: Derivation and validation of an algorithm to predict transitions from community to residential long-term care among persons with dementia—A retrospective cohort study
Source: PLOS Digit Health. 2024 Oct 18;3(10):e0000441. doi: 10.1371/journal.pdig.0000441 (PMC11488705; doi:10.1371/journal.pdig.0000441)
Supplement: Attachment — Submitted filename: Response to Reviewers Comments_PLOS DH.pdf [file pdig.0000441.s010.pdf]

<u>Response to Reviewers</u>

Derivation and Validation of an Algorithm to Predict Transitions from Community to Residential Long-term Care among Persons with Dementia – A Retrospective Cohort Study

We thank the PLOS Digital Health editors and the reviewers for considering our manuscript and for facilitating the review process. These comments are very insightful and have helped us improve the paper. We have addressed the editors' and each reviewer's comments below, with our response in blue following each comment.

<u>Reviewer 1 Comments:</u>

This study utilized data from over 100,000 older adults assessed by RAI-HC between 2010 and 2017 to develop and validate a model predicting LTC admissions among individuals with dementia. Both dementia and LTC admission were determined using validated algorithms, and the TRIPOD reporting guideline was followed. The algorithm was developed using self-reportable items to support patient-oriented risk communication. Overall, the study was well-conducted and reported. Here are some minor comments:

We thank the reviewer for their support for our study and their constructive feedback to improve our paper.

1. The tool is designed to supplement the MAPLe score, an established score for assessing priority levels for LTC admission. The authors stated that 80% of home care clients living with dementia were classified into the two highest MAPLe categories. However, this number was based on a study published in 2014. To more robustly establish the superiority of the currently developed score, the performance of the MAPLe algorithm in the derivation and validation data should be assessed and compared.

We have indeed performed some additional analyses to examine the performance of the MAPLe score in predicting time-to-LTC entry. The c-statistics of MAPLe on the validation cohort is 0.59 (compared to 0.71 for our model); the calibration plot for MAPLe is available in Supplementary Materials 1 – Additional Results (S1. Figure 2). In our submission and manuscript, we had purposely decided against comparing the performance of MAPLe to the performance of our algorithm since this comparison would not be fair. The MAPLe was never designed to be specific to the dementia population, nor is it a prediction tool. MAPLe was designed to be a decision support tool and used to prioritize clients needing both community- and/or facility-based services and to assist with allocation of resources. We did not want to misrepresent MAPLe as a predictive algorithm, nor imply that it is not useful or valid for its intended use cases. The motivation for the development of our algorithm comes from our belief that a more specific and nuanced tool is currently lacking for the dementia population – which constitutes a substantial proportion of individuals seeking long-term care placement.

2. It would be helpful for international readers if some background information on the LTC system in Canada could be provided. The authors noted that the data may include individuals who have already applied for LTC. Could you provide information on the median waiting time

and how eligibility is determined? Additionally, how might this subset of individuals affect the results, as they are likely to be placed earlier and could potentially inflate accuracy?

We have added some additional information (lines 16-19; 48-53) around the context for LTC placement in Canada. In short, anyone who intends to apply for LTC placement must be first assessed using the RAI-HC. Anyone who didn't seek out LTC but are receiving home care would also receive the RAI-HC, which will prompt the care team to consider LTC if the individual's care needs cannot be met in the community. Thus, the RAI-HC assessment is a necessary step for all individuals before being placed in LTC. As such, there would be no bias in our cohort regardless of intention.

Median wait time for LTC placement in Ontario varied over the years. Relevant to our cohort capture years of 2010 to 2017, the median wait time for LTC placement were: 126 days in 2013-14, 110 days in 2014-15, 113 days in 2015-16, 133 days in 2016-17, and 146 days in 2017-18.[1] Median wait time was not available prior to 2013.

3. The authors note that including CPS > 2 was shown internally to increase the number of dementia cases captured by 8%. Is there a reference available for this? Additionally, please confirm whether it is CPS = 2 or CPS ≥ 2.

We thank the reviewer for catching this oversight, and have clarified in the manuscript that it was a CPS score of equal or greater than 2. Unfortunately, we cannot provide a reference for the increased capture of cases using this definition as the validation work to show increased capture was done for an internal (and therefore unpublished) report for the Public Health Agency of Canada. PHAC is actively using this definition to revise their projection of dementia burden – this work will likely become publicly available in the future.

4. Age was modeled using restricted cubic splines with 5 knots. Could you provide the rationale for not including age in years in its original format?

Restricted cubic splines (RCS) are piecewise cubic functions that are smooth at the knots and restricted to be linear in the tails. We placed the knots at fixed quantiles (i.e., the 5th, 27.5th, 50th, 72.5th and 95th percentiles) of the distribution of age. RCS is a recommended method for modelling continuous variables when linearity is not met and has several advantages.[2,3] First, it allows for greater flexibility in the relationship between the continuous variable and the outcome. Additionally, like polynomials, it produces smooth dose-response curves. However, unlike polynomials with high-order terms which can lead to extreme results under extrapolation, RCS always produces a linear extrapolation. Finally, the placement of knots provides additional degrees of freedom, which can be used either to reflect features of the relationship being modeled or to improve the fit.

5. How was collinearity defined in this study? Was there a pre-specified cutoff value used?

Thank you for catching this, we have modified the manuscript to clarify that collinearity was assessed using the SAS VARCLUS procedure.[4] In brief, VARCLUS, or variable clustering, is used for assessing collinearity and redundancy by quantifying the associations between predictors while allowing for general nonmonotonic relationships. This is done by using Hoeffding's D as a similarity measure for all pairs of predictors.

Reviewer #2:

Using a number of administrative data sources in Canada, the authors develop and validate a prediction model for transitioning to long-term care amongst older adults with dementia. Given that the disease course for adults with dementia is highly variable, often with significant resource implications, there is a clear need for prognostic tools both for patients/caregivers and providers/health systems. I think the work here is also well-motivated by highlighting the deficiencies of the MAPLe tool that is currently being used in Canada. Statistically, the authors correctly employ an approach that accounts for the competing risk of death, evaluate both discrimination and calibration, and while there is a not a true external validation, the design of using a past dataset to predict the future is how such models would be deployed in practice. Overall, I think the predictive performance of the model is about what I'd expect, good, but not great. That's less of an issue of the data available or modeling approach, but just reflects the reality that predicting shorter term outcomes in these types of populations is challenging because a component of what occurs is inevitably random/stochastic and not predictable. Below I offer some suggestions for improvement related to potential comparisons.

We thank the reviewer for their support and their recognition of the strengths of our study, and their valuable feedback to improve our paper.

Major Comments

1. I think the paper would strengthened by evaluating the performance of the predictive model for 1-year mortality of Deardorff et al. (JAMA Intern Med. 2022 Nov 1;182(11):1161-1170. doi: 10.1001/jamainternmed.2022.4326.) in the validation cohort. While that is designed for a different outcome in individuals with dementia, there is a natural question of whether we need yet another model, or perhaps those at high mortality risk are also generally the same individuals at high risk of transitioning to long-term care? A risk equation derived specifically for long-term care transitions should perform better, but the obvious question is by how much?

We thank the reviewer for bringing the algorithm developed by Deardorff et al. to our awareness. It is a very good model and will help inform many of our future studies. However, we cannot validate the Deardorff et al. algorithm using our data since the many of the predictors included in their model, such as smoking status, race, and BMI, are not available in Ontario's (and other

Canadian provinces') health administrative databases/clinical assessments. While we acknowledge that differential predictors across jurisdictions may reduce the generalizability of our algorithm, we still believe that our algorithm will have wide use cases since the RAI-HC assessment is widely implemented in many Canadian provinces and internationally. Similar to many other algorithms developed using the RAI data, our work can lead to the creation of flags embedded in the interRAI assessment systems for routine monitoring and timely alerting of need for transitions. We also have plans to implement it as a web calculator, as explained below.

2. The comparison to Deardorff et al. also raises the issue of model availability. Their model is available as a web calculator on ePrognosis.org. As is stands, while someone could get most of the way if they wanted to evaluate this model in a different cohort, there are issues with re-creating the cubic splines with age and not having a way to access the non-parametrically estimated baseline component of the Fine-Gray model. It would be ideal to provide a R model object that could be used for prediction, perhaps in tandem with a R/Shiny web calculator. I do recognize that both of those outlets implicitly need to have the underlying dataset available/stored, which is probably an issue given the provided statement on data sharing from ICES. There may not be a solution here, but I strongly believe its important to promote risk stratification tools that can be openly evaluated, locally calibrated, etc.

We completely agree with the reviewer and also believe strongly in promoting and implementing prediction tools in a transparent and reproducible manner. We also acknowledge the challenges in data sharing in light of the ICES data sharing agreement. We also want to express our intention to implement this algorithm as a web calculator on the Project Big Life (PBL) website – a platform that has already hosted several of our algorithms and has garnered millions of use cases around the world. The process of implementing the algorithm (including web design, wording of questions, reducing the model to be parsimonious etc) will be rigorously discussed and tested with implementation scientists and patient/caregiver partners, as we have done for past algorithms. While we cannot make the data publicly available, we will publish on Github the codes for the web calculator at the launch of this algorithm as a web calculator, as we have done for previous algorithms. We believe that these future efforts will ensure that our algorithm will generate wide reach and impact and be as open and reproducible as possible.

3. The RAI-HC assessment provides an impressive wealth of data, particularly around functional status which is often not available in claims or the electronic health record. But, a natural question is whether you need all the variables included in the current model, or would a more parsimonious model perform about the same? This is an important issue because in the US, for example, I doubt we'd ever have this breadth of information available clinically. I think a way to approach this would be to compare to a simplified model that mimics what would be available in the electronic health record. So, something like, don't know education and marital status, or place of residence, have ADL/IADL but not the other assessments, and then just have information on comorbidity and medications?

We thank the reviewer for recognizing the strength of the RAI data. Indeed, data availability (or the lack of) is always an issue for algorithm development and generalizability. We first want to share that the interRAI assessments are used in 24 states in the US – though not as systematically given the differences in healthcare systems in the US. We also agree that reducing the model into a more parsimonious one would be important, particularly when we implement the model as a web calculator or for specific types of clinical use. For many of our previous algorithms implemented as web calculators on the PBL website, we reduce the number of predictors in the model – the extent of the parsing and decisions on which factors to keep/eliminate is not only based on the strength of the predictor, but also on what is most relevant/reportable for the use case and users involved. For several previously created algorithms, we are in the process of creating specific models for the different implementations use cases, in consultation with caregiver/patient/clinician advisors. We will do the same once this algorithm is published. Additionally, we have received funding to externally validate and create variations of several predictive algorithms (including this one once it is published) using different data sources (e.g., a suite of longitudinal studies on aging, starting with the Canadian Longitudinal Study on Aging), different methods (e.g., machine learning approaches), and including additional/different sets of predictors (e.g., laboratory testing data). Through these efforts, we will produce a baseline model with the minimal sets of universally-available predictors needed to achieve sufficient model performance, then examine how much the model can be improved by adding additional predictors. We look forward to pursuing these efforts.

Minor Comments

1. Abstract. I would add some result that quantitatively reflects calibration to the results section.

We thank the reviewer for this suggestion, and have modified the Abstract with quantitative calibration indices.

2. I believe information on race or ethnicity is not collected in the data assembled at ICES, correct? Presumbly this reflects a large majority white population, so it is unknown how this model would perform in more diverse populations, whether the model is fair, etc. I would add a thought along these lines to the limitations.

Indeed, race is not collected in Ontario's health administrative data. However, Ontario is actually the most populous and has the second highest percentage of visible minorities among Canadian provinces. 34.3% of the province's population belongs to a visible minority group and 2.9% are from Indigenous populations.[5] Ontario is also the most religiously, culturally, and linguistically diverse province in Canada. For example, Ontario houses almost half of all newcomers to Canada and has the highest percentage of Muslims (6.7%) and Hindus (4.1%), and other non-Christian religions (5.8%) in Canada.[6,7] This level of diversity would be considered high among

most English-speaking countries/jurisdiction, where the tool could be applicable for. As such, we believe that the lack of diversity is not an issue for our algorithm.

3. Another limitation that should be acknowledged is that the whole analysis rests on a dementia ascertainment mechanism that almost certainly underestimates the community-burden of dementia, and likely concentrates on those at later stages of the disease. I think the algorithm employed is as good as one can do from administrative data, but there is no way to overcome the reality that dementia tends to be under-coded, and when it is, it is later stages of the disease. Could cite work such as Tsoy et al. (JAMA Neurol. 2021;78(6):657–665. doi:10.1001/jamaneurol.2021.0399) that generally demonstrate this issue.

We thank the reviewer for this suggestion, and have added this as a limitation in the manuscript.

4. Lines 117-118. I would add a brief sentence here that summarizes what variables tended to be missing the most along with frequency.

We thank the reviewer for this suggestion, and have modified the manuscript accordingly (lines 121-124)

5. Lines 129-130. "Finally, model coefficients for algorithm implementation were generated based on the total cohort."

We thank the reviewer for this suggestion, and have modified the manuscript accordingly

6. Line 162. "Subdistribution hazard ratio (sHR) estimates…." I typically use this labeling of sHR to differentiate that you're using Fine-Gray and not a cause-specific Cox model.

We thank the reviewer for this suggestion, and have modified the manuscript accordingly

7. I would add the histogram of the predicted risk distribution in Figure S2 to the calibration plot shown in Figure 2.

Thank you for this suggestion. We had previously layered the calibration plot onto the predicted risk distribution, but a few colleagues/reviewers found it confusing and suggested that we separate them out. We do believe that the risk distribution would be helpful for interpretation of the calibration plot, and have now put Figure S2 and Figure 2 side by side as Figures 2A and 2B. We hope this would be helpful for readers.

References:

1. Health Quality Ontario. Wait Times for Long-Term Care Homes. Accessed November 18, 2020. https://www.hqontario.ca/System-Performance/Long-Term-Care-Home-Performance/Wait-Times

2. Harrell FE. *Regression Modeling Strategies*. Springer New York; 2001. doi:10.1007/978-1-4757-3462-1

3. Gauthier J, Wu Q V., Gooley TA. Cubic splines to model relationships between continuous variables and outcomes: a guide for clinicians. *Bone Marrow Transplant*. 2020;55(4):675-680. doi:10.1038/s41409-019-0679-x

4. SAS Institute Inc. The VARCLUS procedure. Published online 2014. http://documentation.sas.com/doc/en/statug/15.2/statug_varclus_toc.htm

5. Statistics Canada. Census Profile, 2021 Census of Population. Statistics Canada. Published November 15, 2023. Accessed May 16, 2024. https://www12.statcan.gc.ca/census-recensement/2021/dp-pd/prof/index.cfm?Lang=E

6. Government of Ontario. Newcomers. Government of Ontario. Published August 3, 2023. Accessed May 16, 2024. https://www.ontario.ca/page/newcomers

7. Government of Ontario. *Creating a More Inclusive Ontario: Diverse Populations Addendum*.; 2021. Accessed May 16, 2024. https://files.ontario.ca/msaa-diverse-populations-addendum-en-2021-01-01.pdf
