# Supplementary material for: Derivation and validation of an algorithm to predict transitions from community to residential long-term care among persons with dementia—A retrospective cohort study
Source: PLOS Digit Health. 2024 Oct 18;3(10):e0000441. doi: 10.1371/journal.pdig.0000441 (PMC11488705; doi:10.1371/journal.pdig.0000441)
Supplement: Attachment — Submitted filename: Response to Reviewers_PLOS DH_July 15, 2024.pdf [file pdig.0000441.s011.pdf]

Response to Reviewers – July 15, 2024

Derivation and Validation of an Algorithm to Predict Transitions from Community to Residential Long-term Care among Persons with Dementia – A Retrospective Cohort Study

We thank the PLOS Digital Health editors and the reviewers for further reviewing our manuscript. We have addressed the additional comments from Reviewer #2 below, with our responses in blue following each comment.

Reviewer #2: I appreciate the author's thoughtful edits and responses to my initial critique. I have only a few minor additional suggestions.

We thank the reviewer for their additional feedback – they are very helpful in helping us improve our manuscript.

With respect to model dissemination, I think it would be help to add a brief paragraph, perhaps titled "Future Directions", right before the Conclusion. I would add much of what was said in response to my initial bullet # 2. Even if this model is not immediately available now, I think it's important to point readers to the PBL website, and to mention your intention to pursue further external validation and also pursuing user engagement before deploying this model. Don't need a lengthy discussion, but this will help a reader know where to look for future progress.

This suggestion is really helpful – we have added the Future Directions paragraph to the manuscript (lines 243-252)

With respect to my minor comment #2, I'm curious if the provided population statistics are restricted to older populations? For example, in the US, while there are ever growing populations of Blacks, LatinX, Asians, etc., the population is still very much majority white in the age range where dementia tends to occur (roughly 70 or 75 years plus). I'm going to guess it's similar in Ontario. I do think a qualifier needs to be added to the limitations that mentions that generalizability to broader populations is unknown, and is an important topic for future development of the algorithm.

Thank you for bringing this to our attention – the statistics provided in our previous response indeed did not restrict to an older population. We have now checked the ethnicity of older adults in Ontario in 2016: according to Statistics Canada (link below), 17.6% of older adults (65+) in Ontario were from racialized groups in 2016. This is indeed a lot lower than the 34.3% in our previous response, though still highest among all Canadian provinces.

https://www150.statcan.gc.ca/n1/pub/71-607-x/71-607-x2022012-eng.htm

As suggested by the reviewer, we have added a qualifier in the Limitations section (line 239) to state that generalizability to a broader population is unknown. One of our future efforts will be to

externally validate this algorithm.

Abstract, Conclusions and Implications. Perhaps rephrase as “We developed an algorithm to predict to time LTC entry among individuals living dementia that improves upon an existing tool used for prioritization.” Well-performing is a bit of a nebulous term here.

We agree with the suggestion and have modified the text in the Abstract to remove “well-performing”.

Lines 213-222. One other thought that you could mention is that the calibration issue may also reflect artificially ending the time horizon at 1 year. If many of these individuals ended up getting placed in LTC in say the following year, the model isn’t “wrong” for assigning higher risk to these individuals. Certainly see this type of pattern in trying to predict mortality, where short term outcomes are very, very hard to calibrate, but as one moves out to 2 or 3-year mortality, outcomes tend to become much more predictable and one gets better calibration.

We really appreciate this suggestion and have added this consideration to the Discussion section (lines 213-218).
